# The incidence of periungual desquamation and thrombocytosis in Kawasaki disease and the importance of systematic observation in the subacute phase

**DOI:** 10.3389/fped.2024.1384015

**Published:** 2024-07-08

**Authors:** Beom Joon Kim, Arum Choi, Sukil Kim, Ji-Whan Han

**Affiliations:** ^1^ Department of Pediatrics, College of Medicine, Catholic University of Korea, Seoul, Republic of Korea; ^2^ Department of Radiology, College of Medicine, The Catholic University of Korea, Seoul, Republic of Korea; ^3^ Department of Preventive Medicine and Public Health, College of Medicine, Catholic University of Korea, Seoul, Republic of Korea

**Keywords:** desquamation, fever, incomplete Kawasaki Disease, intravenous immunoglobulin, Kawasaki Disease, Kawasaki-like Disease, subacute phase, thrombocytosis

## Abstract

**Background:**

Periungual desquamation and thrombocytosis are characteristic of the subacute phase of Kawasaki disease (KD). However, accurate observations of periungual desquamation and thrombocytosis are lacking.

**Methods:**

This retrospective study included patients with acute-phase KD who received treatment at seven affiliated university hospitals in Korea between 2015 and 2017. Data were extracted from an anonymized registry established by the Korean Society of Kawasaki Disease. We investigated whether the findings of patients observed according to a set protocol until the subacute stage (group I) were different from those of patients observed without the use of a protocol (group II).

**Results:**

A total of 879 patients with KD were included in the analysis. Periungual desquamation was observed in 85% and 12.7% of patients in groups I and II, respectively. Thrombocytosis was observed in 76.7% and 44.7% of patients in groups I and II, respectively. Furthermore, compared to the initial test, the platelet counts of patients increased 100% and 67.9% in group I and II, respectively. When incomplete KD was defined only by the main symptoms during the acute stage and the diagnostic criterion of periungual desquamation during the subacute stage was excluded, the significant difference in the incidence of incomplete KD between groups I and II was no longer apparent.

**Conclusion:**

Performing regular and detailed observations has resulted in a higher incidence of periungual desquamation and thrombocytosis during the subacute phase of KD than those reported in recent studies. This indicates that until now, we have been neglecting the observation of symptoms and signs during the subacute phase. Regular monitoring during this period can also aid in differentiating suspected cases of KD and facilitate appropriate follow-up of complications.

## 1 Introduction

Kawasaki disease (KD) is an idiopathic and systemic febrile vasculitis that primarily affects children younger than 5 years; additionally, it is the most common cause of acquired heart disease in developed countries (1). Untreated KD can lead to complications such as coronary artery aneurysms (2). Therefore, an accurate diagnosis, timely treatment, and ongoing follow-up examinations are crucial (3). Complete KD is diagnosed based on fever lasting more than 5 days and the presence of at least four of the five main clinical criteria (3). However, the occurrence of incomplete KD, which does not meet the criteria for major symptoms, has increased, resulting in a delayed diagnosis of KD and an increased frequency of coronary artery complications (4, 5).

During the subacute phase of KD, which occurs 2–3 weeks after the onset of fever, distinctive clinical features are observed, including periungual desquamation of the skin and an increased platelet count (3, 6, 7). Previous studies have reported that periungual desquamation occurs in approximately 70%–98% of patients with KD; however, its exact mechanism and association with disease severity remain unclear (8, 9). An increased platelet count has been considered a positive and reactive phenomenon associated with inflammation; however, recent evidence has suggested that it is associated with an increased risk of thrombosis caused by platelet activation (10).

Periungual desquamation and thrombocytosis are important clinical indicators that confirm KD, and they are particularly helpful for distinguishing between KD-like diseases and incomplete KD. These symptoms also signify the transition from the acute phase to the subacute phase of KD, which is a clinically significant period characterized by the initiation of coronary artery abnormalities and an increased risk of thrombosis.

However, the awareness of the importance of symptoms and signs during the subacute phase is insufficient. The early diagnosis and treatment of KD are important for the reduction of coronary artery complications (11). Therefore, the acute stage of KD has been the main interest of those in the medical field, and their interest in the long-term prognosis is often limited to patients diagnosed during the acute stage. There have been few studies of periungual desquamation and platelet counts during the subacute phase. Additionally, some studies have reported that the development of periungual desquamation is significantly infrequent (12).

We believe that this perception is attributable to the lack of appropriate assessments rather than changes in disease characteristics. Therefore, we aimed to investigate the actual incidence rate and clinical significance of periungual desquamation and thrombocytosis in patients with KD based on the use of a protocol and regular monitoring of patients during the subacute phase of KD.

## 2 Methods

### 2.1 Study design and population

We conducted a retrospective study of patients with acute-phase KD who received treatment at seven affiliated hospitals of the Catholic University of Korea between January 1, 2015 and December 31, 2017. Data were extracted from an anonymized registry established by the Nationwide Kawasaki Disease Epidemiological Survey conducted by the Korean Society of Kawasaki Disease from 2015 to 2017. The Korean Society of Kawasaki Disease conducted the survey via postal mail and email. The extracted data included the patients' demographic data and history, all symptoms and signs at the time of the diagnosis of KD, treatment method during the acute phase [all treatments including intravenous immunoglobulin (IVIG) and aspirin], treatment after discharge, laboratory test results during the acute and subacute phases, serial echocardiography and other imaging test results, coronary artery complications, and coronary artery size (maximum internal diameter). Patients with insufficient data were excluded, and duplicate data of patients with the same birthday, sex, and KD onset date were excluded because of the possibility of interhospital transfer.

#### 2.1.1 Diagnosis and definitions

KD was diagnosed according to the 2004 American Heart Association guidelines, and incomplete KD was diagnosed when fewer than four major symptoms included in the diagnostic criteria were present (13). Refractory KD was defined as fever (38°C or greater) persisting 36 h after completion of the first IVIG dose or when second-line treatment was required for KD. Coronary artery anomalies were diagnosed according to the criteria of the Japanese Ministry of Health, Labour, and Welfare after measuring the internal diameter of the coronary arteries (14).

#### 2.1.2 Evaluation protocols for KD

At one of the seven institutions of the Catholic University of Korea, all patients were evaluated using a specific protocol to determine the presence of periungual desquamation and platelet counts during the subacute phase. Observations of clinical symptoms and laboratory tests were sequentially performed at the time of admission (usually 4–5 days after onset of fever), 24 h after IVIG administration, and 7 days after IVIG administration. In particular, the presence of periungual desquamation was determined 7 days after IVIG administration, data were recorded in the medical records, and photographs were obtained. At the other hospitals, laboratory tests were not always performed during the subacute stage. Although they asked most patients with KD about the presence of periungual desquamation in the subacute phase, it was not directly evaluated regulary or recorded in the medical records, and photographs were not obtained.

#### 2.1.3 Group categorization for comparative analysis

We classified the patients into two groups based on the methods used to observe findings in the subacute phase. Group I consisted of patients whose subacute symptoms and laboratory tests were evaluated following a consistent protocol, whereas Group II included patients whose subacute phase findings were documented without a standardized protocol.

To compare the characteristics at the time of hospitalization in the two groups, we compared the patients' demographic data, acute KD symptoms, laboratory tests, and treatment results.

We compared the rates of thrombocytosis and periungual desquamation observed at hospitals where the platelet count and desquamation during the subacute phase were confirmed using a consistent protocol (group I) and those observed at other hospitals that did not use any protocol (group II).

Additionally, we sought to assess the impact of differences in the observation of periungual desquamation between the two groups. We examined how the diagnosis of incomplete KD varied when periungual desquamation was included vs. excluded as a diagnostic criterion.

### 2.2 Statistical analysis

Data were analyzed using R version 4.0.0 (R Foundation for Statistical Computing, Vienna, Austria), and significance was set at *P* < 0.05. During the descriptive analysis, we calculated the frequencies and percentages of categorical variables and means and standard deviations of continuous variables.

Before comparing the characteristics of the groups, we assessed the distribution of the continuous variables to determine the appropriate use of parametric or nonparametric statistical tests. For normally distributed data, we applied Pearson's *χ*^2^ test with Yates' continuity correction for categorical variables and Student's *t*-test for quantitative variables to compare means between the two groups. Fisher's exact test was employed for categorical variables when expected frequencies were low, ensuring accurate assessment of associations in small sample sizes. For non-normally distributed data, the Mann–Whitney *U* test was utilized for quantitative variables, providing a nonparametric alternative for comparing medians between groups.

Additionally, a two-sample test of the equality of proportions with continuity correction was performed to compare the proportions of each of the characteristics of coronary artery abnormalities.

## 3 Results

**Figure 1:**
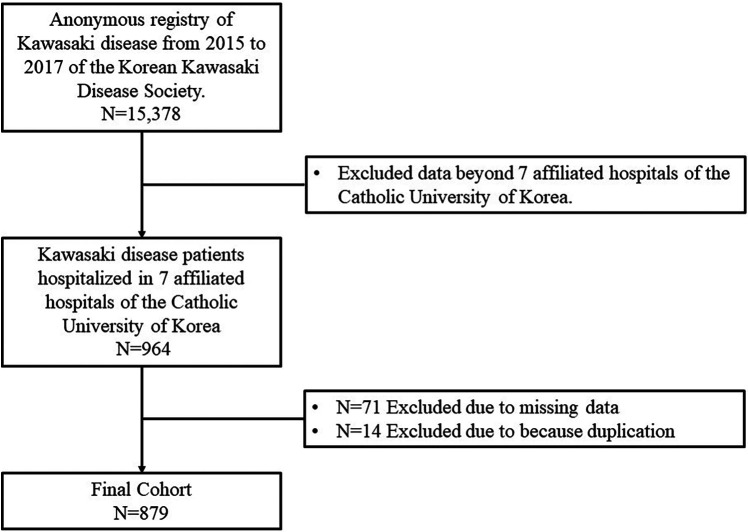
Flowchart of the study population.

Data of 964 patients with KD who received treatment during 2015–2017 at seven affiliated hospitals of the Catholic University of Korea were collected. Among these, 71 patients with incomplete data and 14 duplicate patients were excluded. Finally, 879 patients were included in the final analysis (Figure 1). Sixty patients were regularly observed according to a set protocol to determine the presence of periungual desquamation and thrombocytosis during the subacute phase (group I). The remaining 819 patients were not observed using a set protocol (group II).

The major symptoms and clinical characteristics of patients during the acute phase are summarized in Table 1. Patients in group I were significantly younger than those in group II. There was no difference in sex between groups. The duration of fever did not differ between groups; however, the duration of fever before treatment of patients in group I was significantly longer than that of patients in group II. Among the major symptoms of KD during the acute phase, oropharyngeal changes, erythema, and edema of the hands and feet were observed more frequently in group I. There were no differences in the other symptoms between groups. The rates of incomplete KD diagnosed in the acute phase and IVIG-refractory KD did not differ between the two groups.

**Table 1:** Characteristics of patients with Kawasaki disease at admission.

Variables	Group I (*n* = 60)	Group II (*n* = 819)	*P* value
Age at onset, month	18 (9–43)	28 (12–46)	0.019
Male	31 (51.7)	483 (59.0)	0.330
Total duration of fever, day	6 (5–7)	5 (5–7)	0.279
Pre-treatment fever duration, day	5 (5–6)	5 (4–6)	0.004
Recurrence	5 (8.3)	36 (4.4)	0.392^a^
Clinical features			
Conjunctival injection	59 (98.3)	736 (89.9)	0.101^a^
Oropharyngeal changes^b^	58 (96.7)	672 (82.1)	0.008^a^
Rash	45 (75.0)	628 (76.7)	0.605^a^
Erythema & edema of the hands & feet	49 (81.7)	540 (65.9)	0.036^a^
Cervical lymphadenopathy	29 (48.3)	450 (54.9)	0.492^a^
Incomplete Kawasaki disease^c^	15 (25.0)	291 (35.5)	0.130
IVIG resistant rate	3/60 (5.0)	85/754 (11.3)	0.244^a^

Variables are presented as number (%) or median (interquartile range).

Chi-square test used for count data unless otherwise noted.

^a^ Fisher's exact test used for count data. IVIG, intra venous immunoglobulin.

^b^ Oropharyngeal changes: erythema, lips cracking, strawberry tongue, diffuse injection of oral and pharyngeal mucosae.

^c^ The diagnosis of incomplete Kawasaki disease in this table was evaluated in the acute phase and did not include periungual desquamation.

Table 2 shows the differences in the laboratory and echocardiography results between the two groups. There were no significant differences in most of the results of the blood tests performed at admission, including the platelet count. However, mild hyponatremia was more frequently observed in group I than in group II. The highest platelet count measured by serial blood tests until the subacute phase was observed in group I, and it was significantly higher than any of the platelet counts observed in group II. Significantly more coronary artery complications were observed in group II during the total course.

**Table 2:** Laboratory findings of patients with Kawasaki disease at admission.

Variables	Group I (*n* = 60)	Group II (*n* = 819)	*P* value
Laboratory values at admission			
Leukocytes, × 10^9^/L	13.6 (12.2–15.6)	13.8 (10.6–17.3)	0.572
Neutrophils, %	62.1 (49.4–73.3)	65.5 (53.9–76.7)	0.107
Hemoglobin, g/dl	11.4 (10.7–12.0)	11.4 (10.8–12.0)	0.888
Platelets, × 10^9^/L	365.0 (321.5–420.5)	340.0 (281.0–406.0)	0.143
ESR, mm/h	54.0 (42.0–68.0)	56.0 (40.0–73.0)	0.948
CRP, mg/dl	6.4 (4.7–10.2)	6.6 (3.6–11.1)	0.846
AST, U/L	37.0 (28.0–91.0)	36.0 (26.0–72.0)	0.576
ALT, U/L	34.0 (17.0–106.0)	30.0 (14.0–110.0)	0.370
Total bilirubin, mg/dl	0.3 (0.3–0.6)	0.4 (0.3–0.6)	0.114
Sodium, mEq/L	135 (133.0–136.0)	137 (135.0–138.0)	<0.001
Albumin, g/dl	3.8 (3.6–4.1)	3.9 (3.7–4.1)	0.246
NT-proBNP, pg/ml	560.0 (346.6–754.5)	646.0 (273.6–1,780.0)	0.626
Highest platelets during serial test^c^	550.0 (470.5–627.5)	439.0 (358.5–547.5)	<0.001
Pyuria	33 (55.0)	365 (45.5)	0.195
Coronary artery abnormalities^d^	1/60 (1.7)	78/677 (11.5)	0.015^a^
Dilatation	1/60 (1.7)	67/677 (9.9)	0.006^b^
Aneurysm	0/60 (0.0)	10/677 (1.5)	0.710^b^
Giant aneurysm	0/60 (0.0)	1/677 (0.1)	1.000^b^

Variables are expressed as number (%), mean ± standard deviation, or median (interquartile range).

Chi-square test used for count data unless otherwise noted.

ALT, alanine transaminase; AST, aspartate aminotransferase; CRP, C-reactive protein; ESR, erythrocyte sedimentation rate; NT-proBNP, N-terminal fragment of the prohormone brain-type natriuretic peptide.

^a^ Fisher's exact test used for count data.

^b^ Two-sample test for equality of proportions with continuity correction.

^c^ The timing of the evaluation: subacute phase.

^d^ The timing of the evaluation: total course.

Table 3 shows periungual desquamation and platelet increase during the subacute phase of KD. Periungual desquamation was observed in 51 out of 60 patients (85%) in group I and in 104 out of 819 patients (12.7%) in group II. Thrombocytosis, diagnosed when the platelet count was more than 450,000/μl, was observed in 46 out of 60 (76.7%) and 336 out of 819 patients (44.7%) in groups I and II, respectively. Increased platelet counts compared to those upon admission were observed in all of 60 patients (100%) and 556 out of 819 patients (67.9%), respectively, in group I and group II. The prevalence of subacute periungual desquamation and thrombocytosis were significantly higher in group I, even when only patients with incomplete KD were included in the subgroup analysis.

**Table 3:** Comparison of the proportion of periungual desquamation and platelet increase during the subacute phase.

Variable	Group I	Group II	*P* value
Total patient	(*n* = 60)	(*n* = 819)	
Periungual desquamation	51 (85.0)	104 (12.7)	<0.001
Thrombocytosis	46 (76.7)	366 (44.7)	<0.001
Increased platelets	60 (100)	556 (67.9)	<0.001^a^
Patient with incomplete KD	(*n* = 8)	(*n* = 286)	
Periungual desquamation	5 (62.5)	13 (4.5)	<0.001^a^
Thrombocytosis	7 (87.5)	127 (44.4)	0.025^a^
Increased platelets	8 (100)	180 (62.9)	0.054^a^

Data are expressed as number (%).

Chi-square test used for count data unless otherwise noted.

^a^ Fisher's exact test used for count data.

Table 4 shows the differences in the diagnostic rates of incomplete KD depending on the inclusion or exclusion of periungual desquamation in the subacute phase in the diagnostic criteria for classic KD. When diagnosed without considering periungual desquamation during the acute phase, there was no difference in the incidence of incomplete KD between the two groups. However, when KD was diagnosed including periungual desquamation in the subacute phase, there were more classic KD diagnoses in group I, and the difference in incomplete KD between the two groups was significant.

**TABLE 4:** Proportion of patients diagnosed with incomplete KD Among all KD cases during the acute and subacute phases.

Variable	Group I (*n* = 60)	Group II (*n* = 819)	*P* value
Incomplete KD during acute phase (without desquamation)	15 (25.0)	291 (35.5)	0.130
Incomplete KD during subacute phase (with desquamation)	8 (13.3)	268 (34.9)	0.001

Data are expressed as number (%).

KD, Kawasaki disease.

## 4 Discussion

Our study showed that many cases of periungual desquamation and thrombocytosis were not carefully observed or recorded. The fact that the diagnostic rate of classic KD significantly increased when periungual desquamation was sufficiently observed confirmed that KD may not be accurately diagnosed when periungual desquamation is insufficiently observed.

The subgroup analysis used data obtained from a previously conducted nationwide retrospective study to investigate the recent epidemiological characteristics of KD in Korea. We believe that the numbers of recently reported cases of periungual desquamation and thrombocytosis is markedly lower than those reported in the past. In particular, we aimed to determine why an unusually small number of periungual desquamation cases was reported by a previous nationwide study. Therefore, despite the limitations of the previous nationwide study, which used a retrospective questionnaire survey, we conducted a subgroup analysis using the same data. Furthermore, our study is subject to additional limitations. Data from each hospital were processed based on the individual discretion of physicians, leading to a variety in the quality of data. Additionally, there was a discrepancy in the number of cases between the two groups. Despite these issues, such discrepancies may imply that many clinicians do not directly and closely monitor the subacute symptoms of KD, a concern that our investigation specifically seeks to emphasize.

During the subacute stage of KD, skin peeling occurs on the tips of the fingers and toes at 2–3 weeks after the onset of fever (3). The exact cause of desquamation is unknown, and only a few reports have investigated its mechanism. Gaspari et al. reported that skin changes, such as erythema, that cause desquamation are mediated by interleukin (IL)-2 (15). Thereafter, T-cell activation by heat shock proteins was suggested as the cause of KD and desquamation (16, 17). Activation of the T-cell system also causes periungual desquamation with other diseases (18, 19). It was also hypothesized that sentinel red blood cells are recruited to the epithelial and mucosal borders as an innate immune response, leading to the main signs and symptoms of KD. Accordingly, erythema and edema form on the tips of the fingers and toes, and disassembly begins at the boundary of the adhesion junction (20).

Various desquamation occurrence rates have been reported. When Kawasaki first reported KD, he described it as a febrile illness characterized by desquamation of the fingers and toes, and 49 of 50 cases were confirmed (1). Studies performed before the 1990s reported a high desquamation incidence of approximately 90% among patients of all races (21–23). During the 2010s, studies reported rates between 53% and 68% (9, 24). These studies suggested that the observed incidence of desquamation may be higher because of the lack of established diagnostic and treatment guidelines and the inconsistent use of IVIG at that time. However, two studies published after 2020 reported an incidence rate of 82%, thus making it challenging to conclude a significant decrease in the incidence of desquamation compared to that previously reported (6, 25). During our study, the carefully observed group (group I) had a desquamation rate of 85%, which was not markedly different from the rate of 87% reported in Korea in 1980 (26). One characteristic of previous studies that reported very high incidence rates was the performance of daily observations in the hospital. Because most desquamation studies relied on retrospective record-based research, the reported incidence rates may have varied because of the inherent limitations of retrospective studies. Notably, recent studies that have not primarily focused on desquamation have reported significantly lower desquamation rates, thus supporting this assertion (12, 27, 28).

Thrombocytosis in patients with KD usually appears during the second or third weeks of illness, when fever has subsided and periungual desquamation has begun (29). It is recognized as reactive thrombocytosis triggered by systemic inflammation and regulated by cytokines such as thrombopoietin, IL-6, IL-8, and IL-11. Moreover, there is a correlation between the degree of the systemic immune reaction and the degree of platelet elevation (7, 30). Thrombocytosis mainly occurs secondarily among pediatric patients, with an incidence of approximately 6%–15% (31). Infections have been the primary cause of childhood thrombocytosis, and KD is the second most common cause, accounting for approximately 10% of cases (31, 32). Although thrombocytosis is common with KD, and although thrombocytosis 7 days after the onset of fever is used as a diagnostic clue for incomplete KD, there have been few reports of its exact incidence (3, 10). Burns et al. reported that thrombocytosis was diagnosed in 55% of patients with KD during a 3-week observation period; however, the sample size of 31 patients was small (33). During a recent study, 49.3% of thrombocytosis cases were observed during the acute phase of KD; however, no cases of thrombocytosis were observed during the subacute phase (10). During this study, thrombocytosis was observed in 76.7% of patients whose platelet counts were monitored periodically until the subacute phase (group I), and all of those patients exhibited an increased platelet count compared to that observed during the acute phase. However, the thrombocytosis rate of the control group without periodic monitoring (group II) was 44.7%, which was similar to the incidence rate observed during the acute phase that was reported by a previous study (10).

Our study results revealed that patients in group I, who underwent regular blood tests and careful symptom observation until the subacute period according to a clear protocol, had significantly higher incidence rates of periungual desquamation and thrombocytosis during the subacute period than patients in group II, who were observed without the use of any protocol. A comparison of the two groups showed that patients in group I had a younger median age and lower sodium levels. Reactive thrombocytosis is more common in younger patients than in older patients (31). However, it is noteworthy that there was no significant difference in platelet counts between the two groups at admission, and that the highest platelet counts observed in subsequent serial examinations were significantly different between the groups. Unlike group I, the peak platelet count in group II increased by only 67% compared to the initial blood test, suggesting a lack of observation of platelet counts until the subacute phase.

Periungual desquamation is not correlated with age; however, severe hand desquamation is positively correlated with age (6, 9). Furthermore, although studies have reported varying results regarding inflammatory markers, such as the white blood cell count, band form, C-reactive protein, erythrocyte sedimentation rate, aspartate aminotransferase, and alanine aminotransferase, that indicate the inflammatory state of KD, periungual desquamation is highly likely to be positively correlated with inflammation (6, 9, 24). During our study, no significant differences in the blood test results between groups were observed; therefore, the difference in the occurrence of periungual desquamation could not be explained by these results.

We believe that the low reported rates of desquamation were attributable to the inadequate evaluation of symptoms and signs during the subacute phase. There was no significant difference in the rate of classic KD diagnosed in the acute phase between the two groups. This implied that the initial major symptoms were similar in both groups. Moreover, blood test results and IVIG resistance during the acute phase were not significantly different between both groups, indicating the overall similarity between them. However, after including periungual desquamation during the subacute phase as a criterion for the diagnosis of classic KD, there was significant difference in the occurrence of classic KD between groups.

The primary goal of KD treatment is to quickly resolve acute inflammation and minimize the risk of coronary artery complications (3, 11). However, achieving this not only requires early diagnosis and prompt initiation of therapy but also meticulous observation throughout the subacute phase of the disease. In our study, while the total fever duration was similar between the two groups, the fever duration before treatment initiation was shorter in group II. This indicates that treatment began earlier in group II. However, temporary coronary artery dilation was observed more frequently in group II, with no significant difference in the occurrence of aneurysms. Previous studies have not established a clear correlation between desquamation, coronary anomalies, and thrombocytosis with coronary anomalies. Some papers have reported no association, while others suggest that more severe desquamation may correlate with fewer coronary complications, and increased thrombocytosis may be associated with a higher incidence of coronary complications (6, 10, 24, 25, 34). Our study has limitations in discussing the correlation between desquamation, thrombocytosis and coronary artery complications, and the outcomes of early treatment. However, the objectives of early diagnosis and the initiation of treatment may inadvertently increase the diagnostic rates of incomplete KD and KD-like diseases. In particular, if patients who are suspected of having untreated KD are not carefully observed during the subacute phase, then the diagnosis of KD may be missed.

Through accurate observations and regular testing up to the subacute phase, we have confirmed the precise incidence rates of periungual desquamation and thrombocytosis. Additionally, this has led to an increase in the diagnostic rates of classic KD. However, our discussion on the implications of these findings is based on historical records and past research, not directly from our own study data. To overcome the limitations of our study, further prospective research is needed. Specifically, it is crucial to meticulously examine the relationships between subacute phase symptoms such as periungual desquamation and thrombocytosis, and factors including incomplete Kawasaki Disease, IVIG resistance, coronary complications, and other Kawasaki Disease predictors like NT-proBNP.

## 5 Conclusion

During the subacute phase of Kawasaki Disease (KD), thrombocytosis increases the risks of thrombosis and coronary aneurysms; therefore, appropriate evaluation and preventive treatment are crucial (7). Additionally, periungual desquamation is a typical symptom during this phase and assists in differentiating incomplete KD and KD-like diseases that are untreated during the acute phase (24). We believe that regular and careful observation of symptoms and signs during the subacute phase will prevent missed cases of KD and enable follow-up of complications. However, recent studies have shown that the incidence rates of thrombocytosis and periungual desquamation during the subacute phase are either not well known or exhibit significant variability. This suggests that previous studies may have reported inaccurate results. We confirmed higher incidence rates of thrombocytosis and periungual desquamation among patients who underwent consecutive observations.

## Data Availability

The raw data supporting the conclusions of this article will be made available by the authors, without undue reservation.
