# Corrigendum: The incidence of periungual desquamation and thrombocytosis in Kawasaki disease and the importance of systematic observation in the subacute phase

**DOI:** 10.3389/fped.2025.1564036

**Published:** 2025-03-11

**Authors:** Beom Joon Kim, Arum Choi, Suk-il Kim, Ji-whan Han

**Affiliations:** ^1^ Department of Pediatrics, College of Medicine, Catholic University of Korea, Seoul, Republic of Korea; ^2^ Department of Biomedical Systems Informatics, Yonsei University College of Medicine, Seoul, Republic of Korea; ^3^ Department of Preventive Medicine and Public Health, College of Medicine, Catholic University of Korea, Seoul, Republic of Korea

**Keywords:** desquamation, fever, incomplete Kawasaki Disease, intravenous immunoglobulin, Kawasaki Disease, Kawasaki-like Disease, subacute phase, thrombocytosis

A Corrigendum on The incidence of periungual desquamation and thrombocytosis in Kawasaki disease and the importance of systematic observation in the subacute phase By Kim BJ, Choi A, Kim S-i and Han J-w (2024). Front. Pediatr. 12. doi: 10.3389/fped.2024.1384015

Error in table

In the published article, there was an error in Table 4, “Proportion of Patients Diagnosed with Incomplete KD Among All KD Cases during the Acute and Subacute Phase.” The total number for this group is currently listed as 521, but it should be 819. The corrected Table 4 and its caption appear below.

**Table 4:** Proportion of patients diagnosed with incomplete KD among all KD cases during the acute and subacute phases.

Variable	Group I (*n* = 60)	Group II (*n* = 819)	*P* value
Incomplete KD during Acute Phase (without desquamation)	15 (25.0)	291 (35.5)	0.130
Incomplete KD during subacute phase (with desquamation)	8 (13.3)	268 (34.9)	0.001

Data are expressed as number (%). KD, Kawasaki disease.

The authors apologize for this error and state that this does not change the scientific conclusions of the article in any way. The original article has been updated.

